# Splicing Regulators and Their Roles in Cancer Biology and Therapy

**DOI:** 10.1155/2015/150514

**Published:** 2015-07-26

**Authors:** Maria Roméria da Silva, Gabriela Alves Moreira, Ronni Anderson Gonçalves da Silva, Éverton de Almeida Alves Barbosa, Raoni Pais Siqueira, Róbson Ricardo Teixera, Márcia Rogéria Almeida, Abelardo Silva Júnior, Juliana Lopes Rangel Fietto, Gustavo Costa Bressan

**Affiliations:** ^1^Departamento de Bioquímica e Biologia Molecular, Universidade Federal de Viçosa, 36570-900 Viçosa, MG, Brazil; ^2^Departamento de Química, Universidade Federal de Viçosa, 36570-900 Viçosa, MG, Brazil; ^3^Departamento de Veterinária, Universidade Federal de Viçosa, 36570-900 Viçosa, MG, Brazil

## Abstract

Alternative splicing allows cells to expand the encoding potential of their genomes. In this elegant mechanism, a single gene can yield protein isoforms with even antagonistic functions depending on the cellular physiological context. Alterations in splicing regulatory factors activity in cancer cells, however, can generate an abnormal protein expression pattern that promotes growth, survival, and other processes, which are relevant to tumor biology. In this review, we discuss dysregulated alternative splicing events and regulatory factors that impact pathways related to cancer. The SR proteins and their regulatory kinases SRPKs and CLKs have been frequently found altered in tumors and are examined in more detail. Finally, perspectives that support splicing machinery as target for the development of novel anticancer therapies are discussed.

## 1. Introduction

Alterations in the alternative splicing pattern are essential for cellular development, differentiation, and response to physiological stimuli. However, abnormal splicing events can generate variants that contribute to different types of diseases, including cancer [1, 2]. Normally, the affected genes encode proteins involved in the main biological aspects of cancer cells such as cell cycle control, proliferation, differentiation, signal transduction pathways, cell death, angiogenesis, invasiveness, motility, and metastasis [3].

Alternative splicing offers the plasticity to reshape the proteome. It provides opportunity for the cancerous cells to subvert the production of protein isoforms for the benefit of tumor growth and spreading needs. Many of these processes represent a genomic return to isoforms normally expressed in a tightly controlled manner during development but repressed in most adult cells. Therefore, the regulation of these events in cancer can be understood as a consequence of the disruption of important developmental pathways [4].

The causing mechanisms of changes in the mRNA processing pattern involve both alteration of primary transcript regulatory sequences (*cis*-acting elements) and modifications in the activity of splicing factors (*trans*-acting elements). As the later ones can act in multiple pre-mRNAs, they have the capability of modifying the expression of multiple genes [5] and may then impact widely the cellular splicing pattern. Among the splicing factors that have been shown with abnormal activity in tumors, the SR proteins have received considerable attention [6]. This class of proteins is extensively phosphorylated in their SR domain mainly by Serine Arginine Protein Kinases (SRPKs) and CDC-like kinases, which affect their subcellular localization and splicing activity [7, 8]. When looking at neoplasia, the lack of control in phosphorylation processes has a causative effect on protooncogenes as well as on splicing activity. It is the case of kinases SRPKs and CLKs which have also been found with altered activity in different types of cancer [9, 10].

Therefore, a better understanding of the regulatory mechanisms of these splicing regulatory elements in cancer biology is essential to support the development of new therapies. In this review, key findings on the roles of alternative splicing and its main regulators in tumor biology are discussed. In addition, pharmacological intervention possibilities that can impact the abnormal processing of pre-mRNAs in tumor cells are also examined.

## 2. Splicing Activity in Cancer Related Pathways and Processes

### 2.1. Apoptosis

Eukaryotic cells are constantly exposed to external and internal stress factors that cause damage to the integrity of the cell of their genome and other molecular components. Numerous cellular adaptive strategies involving pathways that control cell cycle and apoptosis were developed during evolution to ensure the organism survival [11]. As cancerous cells display a behavior that normally tries to avoid apoptosis, in various types of tumors the transcripts of a number of genes related to apoptosis are processed abnormally in order to prevent cell death [12, 13].

A well-known example of apoptosis regulator modulated by alternative splicing refers to the* BCLX* gene. It encodes two isoforms with opposite functions, BCL-XL (antiapoptotic) and BCL-XS (proapoptotic) [14]. The overexpression of the antiapoptotic BCL-XL isoform is related to both poor prognosis in acute myeloid leukemia [15] and chemotherapeutic resistance and poor prognosis in breast, prostate, and hepatocellular carcinomas [16–18]. BCL-XS/BCL-XL expression has been shown to be controlled by a number of splicing factors [19–22] as well as by the activity of a long intronic noncoding RNA named* INXS*, which acts by interacting with the splicing factor SAM68 [23].* INXS* induces apoptosis by favoring the expression of the proapoptotic BCL-XS. The BCL-XS was found downregulated in kidney, liver, breast, and prostate human cancer cell lines in comparison to nontransformed cells, consistent with the observation of elevated levels of the antiapoptotic BCL-XL isoform [23].

The proper activity of the apoptosis regulator FAS has been shown to be an important determinant for clinical outcomes and chemotherapy effectiveness [24]. Besides its transmembrane proapoptotic isoform, the* FAS* gene can also be expressed as a soluble prosurvival variant (sFAS) due to the skipping of exon 6 which encodes the FAS transmembrane domain [25, 26]. Associated with poor overall survival and disease-free survival rates, sFAS levels have been found increased in serum of patients with malignant lymphoma and chronic lymphocytic leukemia [27–30]. Mechanistically, a long intronic noncoding RNA known as* FAS-AS1* is involved in sFAS levels control.* FAS-AS1* binds to and sequesters the RNA binding protein RBM5, inhibiting, in turn, exon 6 skipping and reducing sFAS expression. Moreover, it has been shown that when* FAS-AS1* is expressed, the levels of sFAS are decreased which sensitizes lymphoma cells to FAS-mediated apoptosis [31].

**Figure 1:**
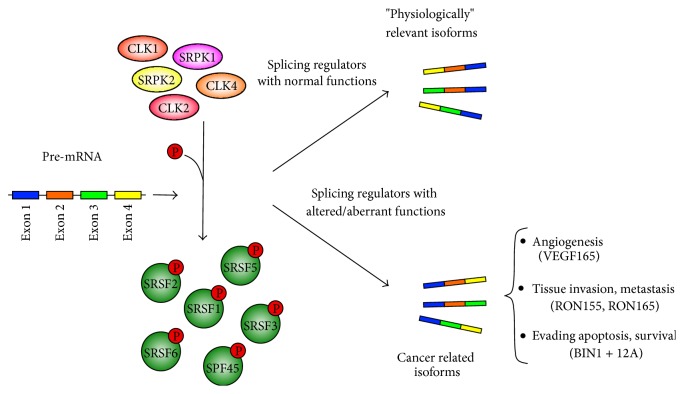
Dysregulation of splicing factors activity in cancer cells. Alternative splicing can generate physiological relevant transcripts in nontumor cells. Alterations in the splicing machinery, such as overexpression or dysregulation of function in regulatory splicing factors, that is, SRPKs, CLKs, or SR proteins, promote angiogenesis, tissue invasion, metastasis, apoptosis evasion, or survival in cancer. These aspects of cancer biology are supported by isoforms that predominate in tumor cells [57, 61–69].

Other splicing events important for apoptosis regulation include the genes* BIN1* and* CASP2*. BIN1 is a tumor suppressor absent in solid cancers including melanoma, neuroblastoma, breast, colon, and prostate cancers [32].* BIN1* gene encodes multiple alternatively spliced isoforms important for DNA repair, cell-cycle control, apoptosis, and membrane dynamics. Some isoforms such as BIN1 +10 and BIN1 +13 have antiproliferative and proapoptotic roles, acting through caspase-independent pathways. In cutaneous T-cell lymphoma, the proapoptotic function of BIN1 isoforms occurs through downregulation of c-FLIP, an important inhibitor of apoptosis mediated by FAS/FASL [33]. However, abnormal splicing of* BIN1* can generate the BIN1 +12A which lacks the tumor suppressor activity [34, 35] (Figure 1).

Considering the* CASP2*, the activity of the RNA binding protein RBM5 increases the synthesis of mRNAs encoding the proapoptotic CASP-2L compared to the antiapoptotic CASP-2S [36]. In ovarian cancer cells, the cisplatin-induced apoptosis was inhibited by CASP-2S overexpression or promoted by its knockdown [37]. The antiapoptotic action of CASP-2S has been shown to be related to its interaction with cytoskeletal membrane associated proteins such as *α*-actinin and fodrin 4. Moreover, CASP-2S has been demonstrated to be responsible for inhibiting DNA damage-induced cytoplasmic fodrin cleavage, independent of cellular p53 status [37].

All these observations reinforce the idea that alternative splicing dysregulation in genes related to apoptosis is an important aspect in cancer research. For additional information about the relationship between apoptosis and alternative splicing, readers are referred to the recent specific reviews [4, 38].

### 2.2. Cell Migration, Adhesion, and Invasiveness

Splicing activity has been found to be important in different steps of metastatic process. It is the case of the cellular alternative splicing reprogramming observed during the epithelial to mesenchymal transition (EMT) in metastatic tumors [39], and the protein isoforms involved in cell migration, adhesion, and invasiveness generated by abnormal splicing [40, 41] (Figure 1). Specific examples are described below.

It has been demonstrated that the CD44 standard isoform (CD44s) plays an important role during EMT in bone breast cancer metastasis [42]. The expression of this isoform has been proved to be controlled by hnRNPM during tumor metastasis, attesting the concept that splicing regulatory networks is a crucial mechanism for cancer phenotypes [43]. Importantly, hnRNPM has been found associated with aggressive breast cancer and correlated with increased CD44s in patient specimens [44]. Mechanistically, ubiquitously expressed hnRNPM can act in a mesenchymal-specific manner to precisely control CD44s splice isoform switching during the EMT observed in tumor metastasis [44].

Other alternative splicing events important during the EMT that occurs in metastatic tumors involve the genes* BCLX* and* RON*. Overexpression of the BCL-XL isoform not only is associated with antiapoptotic function but also is correlated with increased risk of metastasis in breast tumors and multiple myeloma [45]. Moreover, isoforms derived from* RON* alternative splicing, which are involved in the control of cell motility, adhesion, proliferation, and apoptosis, are also related to EMT [46–48]. In this case, isoforms such as RON155 and RON165 are favored by overexpression of the splicing regulator SRSF2, resulting in cell morphology alterations that lead to increased activation in EMT and cell motility [49].

It has also been described that the RNA helicases DDX17 and DDX5 contribute to tumor cell invasiveness by regulating alternative splicing of several DNA and chromatin binding factors, including the macroH2A1 histone. The macroH2A1 splicing isoforms regulate the transcription of a set of genes involved in redox metabolism, such as the extracellular superoxide dismutase 3 (*SOD3*) gene, involved in cell migration [50].

Also, alternative splicing of* KAI1* gene leads to the generation of an isoform lacking exon 7 (*KAI1-SP*) which has been detected in metastatic tissues of gastric cancer patients with poor prognosis [51]. When ectopically expressed, contrarily to the tumor suppressive KAI1, this variant can increase* in vitro* invasiveness and* in vivo* tumorigenicity. These observations suggest that functional differences between these two proteins exist in events such as cell adhesion, spreading, and migration [51]. In ovary cancer, KAI1-SP has been detected with increased expression in metastatic tissues in comparison to primary tumors. Its role in reducing cell adhesion and increasing cell migration was demonstrated to be mediated by integrin ctVp3 [52]. Therefore, splicing activity over the* KAI1* gene leads to the expression of an isoform that favors tumor progression and metastasis [52].

Thus, considering the examples described above it is possible to notice that splicing activity provides critical isoforms for cellular processes that culminate in tumor metastasis.

### 2.3. Angiogenesis

As the tumor mass and size increase, the formation of new blood vessels is required to meet the needs for nutrients, oxygen, and elimination of the diverse metabolic waste. The important role of splicing events in angiogenesis can be fully demonstrated when looking at the control exerted on* VEGFA* gene.* VEGFA* splicing variants are produced due to proximal or distal splicing sites selection at exon 8, resulting in the expression of proangiogenic or antiangiogenic VEGF165 and VEGF165b, respectively [53–55]. Normal tissues can generate both isoforms [55]. Antiangiogenic isoforms have dominant expression in nonangiogenic tissues such as normal colon, whereas proangiogenic isoforms have been found prevalent in cancerous tissues such as colon and skin and in pediatric neuroblastoma [56–58]. Additionally, VEGF antiangiogenic isoforms levels have been found reduced in primary melanoma samples from patients who subsequently developed tumor metastasis compared with those who did not. This data suggests that there is a switch in splicing as part of the metastatic process from antiangiogenic to proangiogenic VEGFA isoforms [57]. This favoring of proangiogenic VEGF165 expression depends on the activity of SRSF1 upon control by the kinases SRPK1/2 [59] (Figure 1).

In colorectal cancer, a novel mechanism for VEGFA isoform expression has been shown to involve the T-cell Intracellular Antigen (TIA-1) activity [60]. A* TIA-1* splice variant encodes for a truncated form called short TIA-1 (sTIA-1). sTIA-1 has been found with elevated expression in colorectal carcinomas and in* KRAS* mutant colon cancer cells and tissues, having its expression increased depending on the tumor development stage. Knockdown of sTIA-1 or overexpression of the full length TIA-1 induced expression of the antiangiogenic isoform VEGFA165b. Interestingly, the increased VEGFA165b translation promoted by TIA-1 is counteracted by sTIA-1, due to prevention of TIA-1 binding to* VEGFA165b* mRNA. sTIA has likewise been demonstrated to impact tumor development in mouse xenograft model by forming bigger, more vascularized, and resistant tumors during treatment with antiVEGF antibodies. Therefore, the finding that aberrant splicing of a translation regulator can modulate differential expression of VEGFA variants certainly adds a new layer of complexity to the angiogenic profile of colorectal cancer and their resistance to antiangiogenic therapy [60].

## 3. Splicing Regulators Related to Cancer: The SR Proteins

Among factors that regulate alternative splicing, the SR proteins family is essential to control and regulate various aspects of mRNA splicing as well as other RNA metabolism events [70–72]. Several studies have reported that changes in the expression or phosphorylation of SR proteins lead to expression of isoforms that stimulate resistance to apoptosis and cell proliferation and migration (Figure 1). These events have been identified in multiple types of cancers such as leukemia, glioma, breast, colon, pancreas, and lung, among others [62–64, 73, 74].

SRSF1 is a SR protein prototype that has been extensively characterized functionally and biochemically. It corresponds to the first splicing factor described as oncogenic and it has been implicated in a number of cancer related mechanisms [65, 75]. For instance, overexpression of SRSF1 in MCF-7 breast cell line has been linked to elevated levels of the isoforms BIN1 +12A (Figure 1) and S6K1-p31 which are involved in decreased tumor suppressor activity and increased oncogenic activity, respectively [61, 65]. Furthermore, SRSF1 has been found to regulate the expression of MNK2a and MNK2b, both splice isoforms of the MAPK pathway component MNK2 [65]. The expression of the isoform MNK2b, for instance, is implicated in the resistance of pancreatic cancer cells to treatment with gemcitabine [76]. Moreover, SRSF1 overexpression has been related to expression of two isoforms of the BCL-2 family proapoptotic BIM, BIM *γ*1 and BIM *γ*2. As they both lack the BH3 domain and the C-terminal hydrophobic regions, proapoptotic functions cannot be performed [77, 78]. Increased SRSF1 phosphorylation induced by hyperactivation of AKT can also result in the production of CASP9 prosurvival isoforms in nonsmall cell lung cancers [79]. In addition, SRFS1 along with the protein SAM68 [80, 81] regulates the expression of the cyclin D1 isoform CD1b which is involved in cell transformation [82, 83]. As previously mentioned (Section 2.3), SRSF1 has also been found to play a crucial role in angiogenesis since its knockdown prevents angiogenesis and tumor growth [59]. Regardless of the examples herein cited, readers may find additional information about the role of SRSF1 activity in cancer in two recently published specific reviews [75, 84].

Other SR protein family members have also been linked to cancer. SRSF3 and SRSF5 overexpression, for instance, have been found oncogenic by means of increasing the levels of the MCL-1 L isoform, which is involved with antiapoptotic response in MCF-7 and MDA-MB-231 cells [85]. Increased SRSF3 expression in colon and ovary cancers has been related to cell transformation and tumor growth maintenance [86–89]. In addition, SRSF6 and SRSF2 have been found engaged in the control of the ratio between the pro- and antiangiogenic VEGFA isoforms VEGFA165 and VEGFA165b, respectively [66, 67, 90, 91]. Also, SRSF2 can control* RON* transcription and splicing due to the exon 11 physical interaction and inclusion [49]. As RON is a protooncogene constitutively active if exon 11 is skipped, when SRSF2 is downregulated it may favor tumorigenesis by generating a prooncogenic RON isoform [49]. In skin cancer, SRSF6 is overexpressed and it can bind to alternative exons of the extracellular-matrix protein* tenascin C* pre-mRNA. This interaction promotes the expression of isoforms related to invasive and metastatic cancer independently of cell type [92].

Based on these examples described above, it is clear that SR proteins have critical roles in tumorigenesis when its normal activity is disturbed.

## 4. Splicing Regulatory Kinases and Their Roles in Cancer

A diverse number of kinases have been reported to transfer phosphate groups to SR proteins [93]. In the next sections, the main players of this context will be analyzed, that is, Serine-arginine Protein Kinases (SRPKs) and CDC-like kinases (CLKs), both responsible for phosphorylating SR proteins* in vivo* [73, 91, 94, 95].

### 4.1. SRPKs

The SRPKs are serine/threonine kinases that specifically recognize and phosphorylate SR proteins at Ser/Arg dipeptide in a processive manner [96–99]. Until now, four members of this protein family have been described in mammalian cells, that is, SRPK1, SRPK1a (spliced form of the previous one), SRPK2, and SRPK3 [100–102]. Whereas SRPK1 is found predominantly expressed in testicles and pancreas, SRPK2 is mainly found in the brain. Both are found moderately expressed in other human tissues such as skeletal muscle and heart and slightly expressed in the lung, liver, and kidney [102]. The expression of SRPK3 seems to be restricted to muscle cells [100, 102] and it has not been linked to cancer so far.

SRPK1 and SRPK2 have been found overexpressed in different types of cancer including breast, colon, pancreatic carcinomas, leukemia, nonsmall cell lung carcinoma, squamous cell lung carcinoma, gliomas, ovary, and hepatocellular carcinoma [62–64, 74, 103, 104]. Increased SRPK1 expression in breast and colonic cancer has been coordinately correlated to the enhancement of tumor grade [63]. Furthermore, targeting SRPK1 using small interfering RNA (RNAi) in cell lines of these two tumors resulted in both increased apoptotic potential and enhanced cell killing after treatment with gemcitabine and cisplatin. These findings seemed to be accompanied by reduced phosphorylation of MAPK3, MAPK1, and AKT [63]. In breast cancer cells, increased levels of SRPK1 and the RNA binding protein RBM4 have been related to apoptosis resistance [105]. In leukemia, SRPK2 overexpression has been shown to result in increased cell proliferation due to SR protein acinus phosphorylation and cyclin A1 upregulation. These data have been complemented by knockdown experiments whose cyclin A1 expression attenuation and cell arrest at G_1_ phase were both observed [64].

Overexpression of SRPK1 and SRPK2 has also been found in lung tumors samples in percentages as high as 92% and 94% for lung adenocarcinoma and 72% and 68% for squamous cell lung carcinoma, respectively [62]. Additionally, SRSF2 overexpression has been shown to mostly accumulate under its phosphorylated form in these patient samples in agreement with the observed overexpression of SRPK1 and SRPK2 [62]. In patients with ovarian cancer, SRPK1 has been found upregulated in 55% of tumor samples.* In vitro* experiments conducted with ovarian cell lines revealed that SRPK1 knockdown can lead to reduced cell proliferation rate, slower cell cycle progression, and compromised anchorage-independent growth and migration ability. Yet, it can lead to a decreased level of phosphorylation of multiple SR proteins, P44/42 MAPK and AKT. Finally, it enhances sensitivity to cisplatin similarly to that observed in breast and colonic cells [63].

SRPK1 has been found upregulated in low-grade gliomas and related to patient prognosis. Moreover, SRPK1 knockdown inhibited glioma cells growth, invasion, and migration in normoxic condition [74]. In clinical samples of hepatocellular carcinoma, SRPK1 has been found upregulated at both mRNA and protein levels [103]. In further* in vitro* and* in vivo* studies, SRPK1 appeared to influence hepatocellular cell growth and malignancy suggesting that SRPK1 plays an oncogenic role and might be a potential therapeutic target in these cancer cells [103].

**Figure 2:**
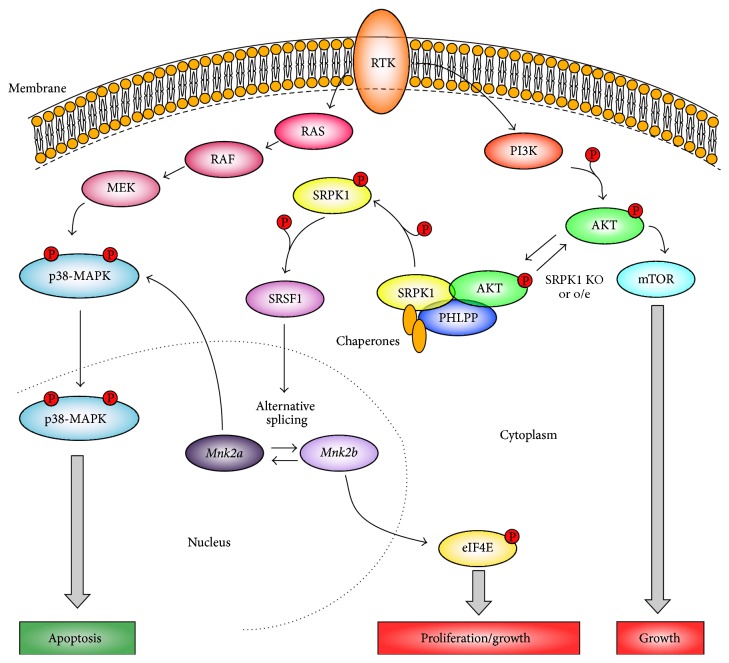
Cellular pathways related to SRPK1 activity. SRPK1 has been connected to important pathways of tumor biology. For instance, it can control alternative splicing events due to the activation of Receptor of Tyrosine Kinases (RTK). In this case, AKT activation can lead to SRPK1 nuclear translocation (not shown), activation of SRSF1, and generation of isoforms such as MNK2b, involved in promoting cell growth and proliferation. On the other hand, MNK2a variant expression, which is disfavored by SRSF1, can promote apoptosis. The phosphatase PHLPP is a key regulator in this process since its activity is necessary for AKT inactivation. Reduced expression of SRPK1 has been shown to decrease PHLPP recruitment to AKT leading to cellular growth increasing. Higher SRPK1 levels, however, may titrate PHLPP away from AKT complex which can also result in AKT/mTOR axis activation. Thus, either overexpression or downregulation of SRPK1 may be oncogenic, explaining why it can be found overexpressed in some tumors but also downregulated in others [106].

Interestingly, it has been demonstrated that depending on the context SRPK1 can act as either oncogene or tumor suppressor [106] (Figure 2). SRPK1 presented tumor suppressor activity since its inactivation in mouse embryonic fibroblasts could induce cell transformation. This phenotype has been related to the impairing of PHLPP recruitment which leads to hyperactivation of AKT by maintaining its phosphorylated form. Furthermore, the overexpression of SRPK1 was observed to be tumorigenic as excess of SRPK1 squelches PHLPP1 and leads to a marked AKT phosphorylation. Therefore, it was concluded that both under- and overexpression of SRPK1 are tumorigenic since both induce constitutive AKT activation [106]. Taken together, these findings could mechanistically explain previous observations that SRPK1 could be found downregulated in some cancer contexts.

### 4.2. CLKs

CLKs comprise a nuclear kinase group that phosphorylates SR proteins. This family is also implicated in the control of splicing and consists of four members,* CLK1*–*CLK4*. While CLK1, CLK2, and CLK4 are ubiquitously expressed, CLK3 is specifically expressed in testicles [107]. The CLKs are characterized by presenting a C-terminal kinase domain with dual specificity, which is closely related to serine-threonine kinases, and an N-terminal RS domain that allows interaction with SR proteins. CLKs colocalize with SR proteins in nuclear speckles. Overexpression of CLKs leads to hyperphosphorylation of SR proteins and induces the redistribution of proteins SR within the nucleus [108]. Although CLKs and SRPKs share common substrates, they have different specificities and act coordinately to regulate splicing properly [109]. For instance, SRPK1 phosphorylates SRSF1 which, in turn, is assembled in nuclear speckles. The release of SRSF1 from speckles depends on phosphorylation by CLK1, also called CLK/STY [9]. CLKs and SRPKs correlated activity can also be demonstrated during the regulation of* VEGFA* splicing. While IGF-1 growth factors and TNF-*α* induce the production of VEGF165 through SRPKs activation, TGF-*β*1 increases the expression of VEGF165b through the activation of CLKs [67].

CLKs have also been related to cancer. For example, CLK1 phosphorylates the alternative splicing factor 45 (SPF45) at eight serine residues (Figure 1). The SPF45 expression is low in normal tissues but high in breast, ovarian, and prostate cancers [68]. In a CLK1 phosphorylation dependent way, the overexpression of SPF45 induces ovarian cancer cells migration and invasion, fibronectin expression, and splicing and phosphorylation of cortactin—a protein that regulates actin polymerization. Another example is the tumorigenic CLK2 which has been found amplified and overexpressed in a significant fraction of human breast tumors [110]. Its downregulation also inhibits breast cancer cell growth and tumorigenesis* in vitro* as well as in a mouse tumor model [110, 111].

## 5. Splicing Activity Related to PI3K/AKT/mTOR and Ras/MAPK Pathways

The PI3K/AKT/mTOR and RAS/RAF/MEK/ERK pathways (Figure 2) are the most frequently impaired signaling pathways in cancer [111, 112]. Alternative splicing machinery dysregulation has been demonstrated to impact the proper physiological signal flow across these pathways, contributing to cell transformation, tumor development, and maintenance [113]. Several examples of abnormal alternative splicing events that affect components of these pathways have been shown in cancerous cells including the tyrosine kinase receptors EGFR, FGFR, INSR, VEGFR, MET, and RON as well as the cytosolic SRC, RAS, and RAF. The alternative splicing events related to these components have been accordingly revised by Siegfried et al. [114]. However, some examples of how alternative regulators can be linked to the abnormal isoform generation or involved in these pathways dysfunction will be discussed below.

As previously mentioned in Sections 3 and 4.1, SRSF1 and SRPK1 have been shown to influence MAPK pathways activity in tumor cells due to their activities as splicing regulators [63, 65] (Figure 2). In addition to the dysregulation of MAPK pathways in colon and breast cancers owing to activity changes in MAP2K1 and MAP2K2, SRPK1 overexpression can also affect the splicing of the MAPK signaling pathway component PYK2 which, in turn, has been associated with cancer development [115]. Considering the regulation performed by SRFS1 on* MKNK2* gene [116],* MKNK2* can be expressed as mRNA two spliced isoforms with differences in the last exons,* MNK2a*, which encodes for a MAPK binding domain, and* MNK2b*, which does not do that [117, 118]. MNK2a interacts and translocates p38*α*-MAPK into the nucleus leading to the activation of target genes, increasing cell death, and suppressing induced transformation by RAS [119]. Alternatively, MNK2b is prooncogenic as it cannot activate p38*α*-MAPK [76] (Figure 2). Thus, downregulation of MNK2a due to SRSF1 activity controlled by SRSF1 constitutes a tumor suppressor mechanism that is lost in tumors such as breast, lung, and colon [119].

Other examples on how splicing activity can affect or be affected by MAPK pathways include the activity of the splicing factor SPF45 and the protein lysyl oxidase-like 2 (LOXL2). SPF45 has been found overexpressed in cancer cells and can be phosphorylated by MAP kinases such as ERKs, JNK, and p38 MAPK in response to phorbol myristate acid (PMA), H_2_O_2_, UV, and anisomycin stimulation [68, 120]. It has been suggested that SPF45 activation via MAP kinases may connect extracellular stimuli to alternative splicing events that may impact cancer. It is the case of the decrease of SPF45-dependent* FAS* exon 6 exclusion, which is a phenomenon observed under ERK and p38 activation. These findings point out that a splicing factor such as SPF45 may be regulated by multiple MAP kinase pathways which can result in alterations in splicing programs relevant to cancer cells.

The LOXL2 protein has also been described as a poor prognosis indicator in human squamous cell carcinomas [121] and as a contributor to tumor cell invasion and metastasis during gastric carcinoma progression [122]. It has been demonstrated that a LOXL2 isoform produced due to lack of exon 13 (LOXL2 Δe13) modulates cancer cell migration and invasion through a different mechanism from that of full-length LOXL2. LOXL2 Δe13 affects MAPK8 expression without affecting the FAK, AKT, and ERK signaling pathways. Differently from the full-length LOXL2, MAPK8 seems to be a downstream component of LOXL2 Δe13, as RNAi-mediated knockdown of MAPK8 results in cell migration blockage promoted by LOXL2 Δe13, but not by the full-length LOXL2 activity [123]. These observations suggest how an abnormal alternative splicing event may affect the activity of MAPK pathway components.

Regarding the pathway PI3K/AKT/mTOR,* S6K1* variants controlled by the splicing factor SRSF1 possess oncogenic properties able to assist breast epithelial cells transformation, motility, and anchorage-independent growth [65]. For example, SRSF1 increases the expression of a shorter oncogenic S6K1 isoform capable of transforming immortal mouse fibroblasts [65]. This small isoform can bind to mTOR and activate mTORC1 leading to an increased 4E-BP1 phosphorylation, cap-dependent translation, and upregulation of the antiapoptotic protein MCL-1 [124].

## 6. Targeting Pre-mRNA Splicing Machinery in Cancer and Its Challenges

Not so long ago, several drugs acting on specific cellular targets started to be approved as anticancer agents. Medicines such as herceptin, gleevec, EGFR inhibitors (gefitinib, erlotinib, and cetuximab), and avastin are now being clinically used to target specific proteins in order to block subcellular pathways relevant to cancer cells [125, 126]. Nevertheless, how patients respond to these drugs is still a puzzle and the answer may rest in the alternative molecules expressed in different individuals when the tumor is under attack during treatment. Thus, although great improvements involving the understanding of cancer mechanisms have been achieved, the treatment and prognosis of tumors remain a big challenge and still require a permanent investigation by academia.

In this review, we discussed the most recent findings regarding how splicing machinery alterations may affect the expression of genes relevant to cancer. As we presented, the findings herein described with focus on the SR proteins and their regulatory kinases, SRPKs and CLKs, highlight the mammalian RNA metabolism as a new source of subcellular targets for the development of anticancer therapies [72]. Despite the availability of a plenty of reports corroborating such idea in the literature, at least two main questions may intrigue scientists in the field: first, are splicing regulators good targets for cancer therapy even if they are expressed in every kind of tissue? Second, how can these drugs be specific for cancer cells?

With our current understanding, these questions cannot be yet fully answered by the available published data. However, some promising experimental results involving pharmacological* in vitro* and* in vivo* inhibition of splicing regulators may help to think over these questions. It is the case of the small molecule inhibitor of SRPK1/2 named SRPIN340. It seems that this compound is effective in blocking angiogenesis and preventing tumor growth in nude mice [59, 127]. Also, SRPIN30 possesses antimelanoma effect* in vitro* and* in vivo* [128]. In addition to this SRPKs inhibitor, pharmacological inhibition of CLKs also seems to be a plausible strategy towards control of tumor growth. This statement can be corroborated taking into account three small CLKs-inhibiting molecules which have been found to modulate* S6K* splicing and suppress breast, lung, and colorectal cancer cell growth* in vitro* [129]. Other CLKs inhibitors that have already been published include the dichloroindolyl enaminonitrile KH-CB19, a potent and highly specific inhibitor for CLK1 and CLK4 [130], and the amino-substituted pyrimidine, a dual specificity inhibitor which targets CLK1, CLK4, and the dual-specificity tyrosine-regulated splicing regulatory kinase DYRK [131]. Furthermore, a 2,4-bis-heterocyclic substituted thiophenes compound has been found to inhibit DYRK1A and 1B, showing a moderate selectivity for DYRK2. Since central nervous system penetration of this compound may occur, it has been believed that it might be used to the development of therapeutic agents against glioblastoma [132].

Even though these reports are encouraging since they suggest novel therapeutic opportunities for fighting cancer, the low pharmacological capacity of some splicing machinery inhibitors (SRPIN340, for instance) has already been noticed* in vivo* [128]. This points to the fact that the search for novel compounds with increased drug-like properties is desirable. Moreover, not all the splicing machinery inhibitors have been evaluated* in vivo* limiting the perception of their real chemotherapeutic potential. Nonetheless, the availability of these* in vitro* and* in vivo* data for the research community* per se *would be considered as an interesting opportunity to guide further studies. The rationalization of these data along with the use of already solved crystallographic structures and deposited in the protein data bank certainly may favor further structure guided efforts to design more favorable substances in the light of the medicinal chemistry knowledge.

Finally, it is not worthless to affirm that cancer treatment is still a great challenge. It is imperative to keep searching for alternative approaches in order to stop the growing list of cancer death cases globally. As a multifactorial disease, cancer demands a better look at patient molecular signatures and predictors in order to pursue an efficient therapeutic regime for each individual who will receive a treatment as specific as the available drug arsenal increases. Thus, cancer control depends on a constant effort toward the discovery of novel and efficient therapeutic strategies [125, 133, 134].

## 7. Conclusions

In recent years, there have been significant advances in research areas that link alternative splicing to cancer. Certainly, there is still a lot to learn about the role of splicing activity within the context of this disease. It is hoped that future studies in the field may favor the development of alternative therapeutic approaches. The recognition of the splicing regulatory kinases SRPKs and CLKs as signal transducers in mammalian cells has opened the doors not only for the understanding of regulatory factors behind abnormal splicing found in tumor cells but also for the development of novel targeting therapies. Thus, based on the investigations herein discussed, it is clear that pharmacological interventions based on regulatory splicing pathways may represent a promising antitumor alternative and should be explored by the scientific community.
